# Mediating role of occupational stress and job satisfaction on the relationship between neuroticism and quality of life among Chinese civil servants: a structural equation model

**DOI:** 10.1186/s12955-020-01295-2

**Published:** 2020-02-19

**Authors:** Wenwen Kong, Yaoyao Yang, Feng Zhang, Hui Wang, Danjun Feng

**Affiliations:** School of Nursing, Shandong University, Wenhuaxi Road 44, Jinan, Shandong China

**Keywords:** Quality of life, Neuroticism, Occupational stress, Job satisfaction, Civil servants, Mediating effect

## Abstract

**Background:**

Knowledge on the quality of life (QOL) of civil servants is limited. Therefore, the aims of this study were to investigate the QOL of civil servants, and examine whether occupational stress and job satisfaction mediated the association between neuroticism and QOL in civil servants from Shandong, China.

**Methods:**

The cross-sectional study included 559 civil servants aged 27 to 60 years from Shandong province in China. Participants completed questionnaires assessing neuroticism, occupational stress, job satisfaction, and QOL. Structural equation modeling (SEM) was conducted to examine the hypothetical model.

**Results:**

Among the civil servants, the average score for QOL was 75.49 ± 14.73. The SEM analysis showed a good fit of the data to the hypothesized model. Neuroticism, occupational stress, and job satisfaction explained 38% of the variance of QOL. Neuroticism was positively correlated with occupational stress and negatively correlated with job satisfaction and QOL. A strong direct effect (− 0.386, *P* < 0.01) and moderate indirect effect (− 0.133, *P* < 0.01) of neuroticism on QOL mediated by occupational stress and job satisfaction were observed. In addition, a direct effect (− 0.197, *P* < 0.01) and an indirect effect (− 0.044, *P* < 0.01) of occupational stress on QOL mediated by job satisfaction were also observed.

**Conclusions:**

Occupational stress and job satisfaction partly mediated the relationship between neuroticism and QOL among Chinese civil servants. Thus, selecting individuals with a low level of neuroticism as civil servants, reducing occupational stress, and increasing job satisfaction may be important measures to improve their QOL.

## Background

Civil servants are those who “perform public duties and have been included in the state administrative system with wages and welfare provided by the state public finance” [1]. As administrators and executors of national affairs, civil servants play an important role in the progress of Chinese modernization. However, to establish a service-oriented government, the management of civil servants has become more stringent. This may put more pressure on civil servants, and thus negatively affect their health. It is reported that the suicide rate among Chinese civil servants is not only higher than that of foreign civil servants, but also higher than that of other professions in China [2]. Civil servants’ health issues can seriously affect their working efficiency and eventually hinder the benign operation of national administrative systems [3]. Therefore, it is necessary to investigate the health conditions of Chinese civil servants.

Quality of life (QOL) is a comprehensive health indicator, and has been widely measured and applied in the field of occupational health. A study of 2492 civil servants in South China found that 41.89% and 43.34% had lower physical and mental health than the general population, respectively [4]. Another study reported that young Chinese civil servants working in the local government had poor QOL, especially in the mental health dimension [3]. Considering the possible poor QOL of Chinese civil servants, research on the risk factors of QOL is particularly important. Numerous risk factors of QOL have been reported for civil servants, including social-demographic factors [4], an unhealthy lifestyle [1, 5], chronic diseases [6], social capital [7], work-family conflict, and coping style [8]. However, few studies have explored the impact of occupational stress [9–12], job satisfaction [13], and personality on the QOL of civil servants. Therefore, this study intended to explore the comprehensive impact of neuroticism, occupational stress, and job satisfaction on the QOL of Chinese civil servants.

Occupational stress is often associated with poor health outcomes and job performance, and has become a serious issue for both employees and organizations. There are several theories explaining how occupational stress occurs. According to the traditional and widely used Occupational Stress Inventory-Revised (OSI-R) model, it is the work environment stressors that lead to the generation of occupational stress [14]. Studies have indicated that Chinese civil servants face many occupational stressors such as a heavy workload and responsibilities, intense job competition, complex interpersonal relationships [15], and more stringent supervision of public opinion [16]. In addition to specific occupational stressors, psychosocial work characteristics are also important causes of occupational stress, and the other two frequently used occupational stress theories described the psychosocial work characteristics. According to the job demand-control (JDC) model [17], occupational stress arises when job control is low and job demand is high. Whereas, the effort-reward imbalance (ERI) model [18] emphasizes that the failed reciprocity between efforts spent (e.g., demands and obligations) and rewards received (e.g., income and esteem) could result in sustained stress reactions [19]. On the one hand, the steeply hierarchical organizations and rule-bound administrative management systems in China increase civil servants’ job demands and reduce their job control. On the other hand, civil servants usually have stable but comparatively low salaries, and their promotion is difficult because of the lack of leadership positions. This may increase the occupational stress of civil servants. Studies have shown that occupational stress directly affects QOL by leading to negative physical and psychological consequences such as migraines, insomnia, anxiety and depression [20–23].

Job satisfaction reflects “whether employees find their employment sufficiently satisfactory to continue in it, either permanently or until they have prepared for greater responsibilities [24]”. It is generally perceived to be directly affected by occupational stress [25], and identified as an important predictor of employees’ QOL [26]. Ibrahim’s study confirmed that nurses in high-workload departments had lower job satisfaction, which was positively related with QOL [27]. Therefore, occupational stress may indirectly affect QOL through the mediating effect of job satisfaction.

Most research on occupational health focuses on work-related factors. However, individual factors, especially personality traits, play an important role as well. A systematic review indicated that personality was a stronger determinant of QOL than socio-demographic and clinical factors [28]. Currently, the Big Five Factor Model (conscientiousness, extroversion, neuroticism, agreeableness, and openness) is the most established model to describe personality. Of these factors, neuroticism is widely considered an important trait affecting health. For example, studies showed that neuroticism was negatively related with QOL [29], and could explain 39% of the variance in psychological health related QOL, and 17–29% of the variance in physical health related QOL [28].

Neurotic individuals tend to identify more stressors and react negatively to unpleasant or threatening environmental stimuli [30]. Therefore, neuroticism is associated with higher occupational stress [31]. Furthermore, neuroticism is negatively correlated with job satisfaction, because it affects the perception and evaluation of the work environment and emotional experience of events at work [32]. In addition, one empirical study also proved that occupational stress partially mediated the relationship between neuroticism and job satisfaction [33].

**Fig. 1:**
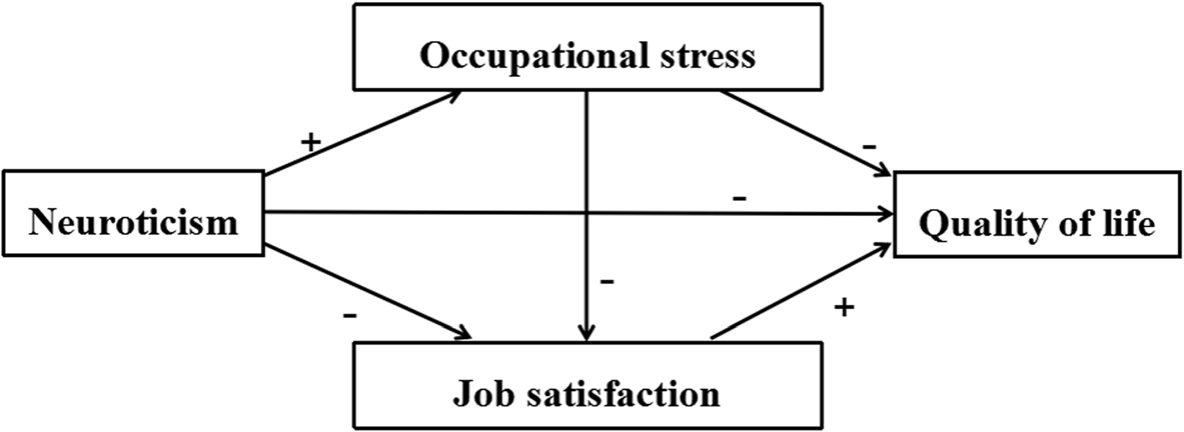
Research framework

Based on the above mentioned existing literature, neuroticism, occupational stress, job satisfaction, and QOL are related and have complex relationships. Although the single effect of neuroticism [34], occupational stress [12], and job satisfaction [13] on QOL have been previously studied, their synergistic effects on QOL and the potential mechanism remain unclear. Therefore, the current study aims to fill this gap. This study hypothesized that neuroticism exerted direct and indirect effects through the mediating role of occupational stress and job satisfaction on QOL among Chinese civil servants. Figure 1 shows the conceptual framework employed in this study.

This study had two purposes. The first was to assess the QOL of Chinese civil servants. The second was to investigate the relationship between neuroticism, occupational stress, job satisfaction, and QOL using the structural equation modeling (SEM) approach.

## Methods

### Study design

A cross-sectional survey was conducted to assess the QOL of Chinese civil servants and explore the relationship between neuroticism, occupational stress, job satisfaction, and QOL.

### Research participants

The participants were civil servants undergoing professional training at the Shandong Academy of Governance from July 2017 to November 2018. Shandong Academy of Governance is managed by the People’s Government of Shandong Province, China, and provides free professional training to the civil servants of Shandong Province. In total, 559 valid responses were received of the 590 questionnaires distributed, representing a response rate of 94.7%.

### Measurements

#### QOL

**Fig. 2:**
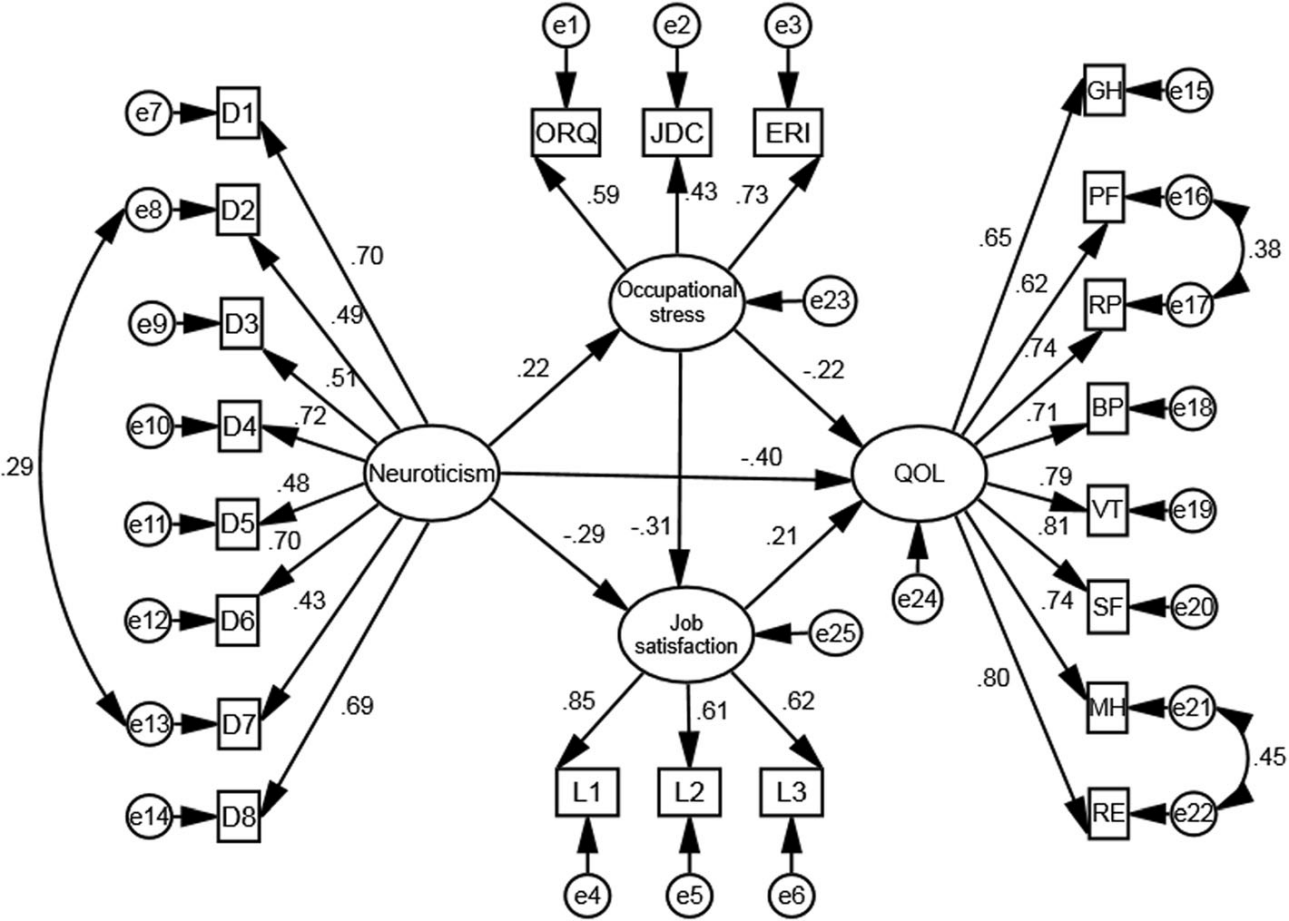
Path diagram for the relationship among neuroticism, occupational stress, job satisfaction, and QOL. All coefficients in the figure are significant at 0.001 level. The variables named D1-D8 are the items of Neuroticism subscale; The variables named L1-L3 are the items of job satisfaction scale; ORQ, Occupational Role Questionnaire; JDC, job demand-control condition; ERI, effort-reward imbalance condition; GH, general health perceptions; PF, physical functioning; RP, role limitations due to physical health problems; BP, bodily pain; VT, vitality; SF, social functioning; MH, mental health; RE, role limitations due to emotional problems

The Short Form-8 (SF-8) Health Survey was conducted to measure QOL, which was derived from the Short Form-36 Health Survey. SF-8 can be completed in one to 2 mins, and can yield scores comparable to the Short Form-36 [35]. The Chinese version of SF-8 was translated and tested by Wang et al., and demonstrated good internal consistency reliability and criterion validity [35, 36]. Therefore, SF-8 has been applied to Chinese occupational groups [37]. SF-8 includes eight items that separately measure eight sub-scales: general health perceptions (GH), physical functioning (PF), role limitations due to physical health problems (RP), bodily pain (BP), vitality (VT), social functioning (SF), mental health (MH), and role limitations due to emotional problems (RE). Subjects responded to each item on a five-point scale. The sub-scale scores can be transformed into standard scores ranging from 0 to 100, with higher scores indicating better health. A standard score was computed using the following formula: standard scores = (actual raw score − lowest possible raw score possible) × 100 / raw score range [1]. Two summary scores were calculated using the weighted sum of the sub-scale scores: the physical component summary (PCS, includes GH, PF, RP, and BP) and mental component summary (MCS, includes VT, SF, MH, and RE). In this study, SEM was conducted to assess construct validity, and the results confirmed that the factor loadings of the eight indicator variables were no less than 0.62 (see Fig. 2), indicating acceptable construct validity. Cronbach’s α for the SF-8, PCS, and MCS was 0.906, 0.818, and 0.883, respectively, indicating satisfactory internal consistency.

#### Neuroticism

The neuroticism personality trait was measured using the Chinese version of the neuroticism subscale from the 44 items of the Big Five Inventory [38]. The questionnaire comprises eight items measured on a five-point scale (1 = strongly disagree; 5 = strongly agree), and the final score is the sum of these items. The construct validity of this questionnaire was confirmed through SEM, and the factor loadings of the eight indicator variables were above 0.42 (see Fig. 2). Cronbach’s α for the questionnaire was 0.819 in this study.

#### Occupational stress

This study combined the OSI-R, JDC, and ERI models to measure professional stress. Therefore, the measurement of occupational stress in the current study was comprised of three parts: occupational roles, JDC, and ERI.

According to the OSI-R model, occupational stressors originating in the work environment influence the perception of work roles [14]. Hence, the first part of occupational stress was assessed using the Occupational Role Questionnaire (ORQ), a sub-scale of OSI-R [14]. The ORQ used in this study was the revised version, which demonstrated good internal consistency reliability and criterion validity for a group of Chinese judges [29]. The revised ORQ includes 22 items and 4 sub-scales: role overload (6 items, an increasing and unreasonable workload), role boundary (5 items, feeling caught between conflicting supervisory demands and factions), responsibility (6 items, responsibility for activities and work performance), and physical environment (6 items, work schedule, working conditions, or feeling personally isolated). As differences in occupation could influence reliability and validity, a confirmatory factor analysis (CFA) was performed to assess the factor structure using AMOS 22.0. One item of the responsibility sub-scale was deleted based on the factor loading of indicator variables (the factor loading was 0.29), and good construct validity (the factor loadings were not less than 0.5) was thus obtained. Participants responded to each item on a five-point scale ranging from “1 (never)” to “5 (always).” A higher score indicated more severe conditions. Cronbach’s α for the questionnaire was 0.868.

The second part of occupational stress was measured according to the JDC model. The model emphasizes that occupational stress occurs when job demand exceeds job control [17]. Two questions were formulated to assess JDC. The first question was: “Is my job demanding of me (for example, intellectual and physical demands)?” The second question was: “Am I in control of my work (for example, I can control the working time, place, progress, objectives, etc.)?” The response range was “1 (very low)” to “5 (very high),” and the second question was reverse scored. Based on the JDC model, occupational stress levels can only be reflected by considering both job requirements and control. Therefore, the JDC score was created using the sum of the two questions with higher scores indicating higher occupational stress.

The third part of occupational stress was measured according to the ERI model where the imbalance between effort and rewards could result in stress [18]. To assess ERI, participants were asked: “Do I pay too much for my work (both physically and mentally)?” and “How much has my work rewarded me (both financially and spiritually)?” The response range was “1 (very low)” to “5 (very high).” The second question was reverse scored. The ERI score was the sum of the two questions, and higher scores indicated a more imbalanced job effort-reward and occupational stress.

SEM was conducted to assess the construct validity of occupational stress using the three scales with ORQ, JDC, and ERI as the first order factors and occupational stress as the second order factor. The results confirmed that the factor loadings for the three indicator variables were no less than 0.43 (see Fig. 2), indicating that the three indicator variables could effectively reflect occupational stress. The final score for occupational stress was the sum of all items of the three parts.

#### Job satisfaction

We developed three items to measure job satisfaction: (1) Overall, I am very satisfied with my job; (2) I regret doing the job; and (3) I would take the same job if given the chance to choose again. The response range was “1 (strongly disagree)” to “5 (strongly agree),” and item 2 was reverse coded. The final score was the sum, and higher scores indicated greater job satisfaction. A CFA was performed to assess the factor structure, demonstrating good construct validity (the factor loadings were not less than 0.6). Cronbach’s α was 0.713.

### Data analysis

The data were analyzed using SPSS (v. 19.0) and AMOS (v. 22.0) software (IBM Corporation, Armonk, New York, USA). Descriptive statistics were performed to describe the socio-demographic factors, neuroticism, occupational stress, job satisfaction, and QOL of Chinese civil servants. T-tests and a one-way ANOVA were conducted to examine the differences in PCS, MCS, and total QOL scores across socio-demographic factors. A Pearson’s correlation coefficient was used to examine the relationships between neuroticism, occupational stress, job satisfaction, and QOL.

SEM is a method for specifying and testing models of linear relationships between observed variables (variables that can be directly measured) and latent variables (variables that cannot be directly measured and represented by multiple observed variables) [39]. SEM can simultaneously test the factor structure of latent variables and the complex relationships among multiple variables, such as direct and indirect relationships. Therefore, SEM was conducted to examine the mediating effect of occupational stress and job satisfaction on neuroticism and QOL in this study. Indirect effects were estimated by bias-corrected bootstrapping (2000 replications). The indirect effect is statistically significant at the 0.05 level if the bias-corrected bootstrap 95% confidence interval (CI) does not include zero [40].

The following indexes were used in the goodness-of-fit tests for the model: the normed Chi-square (χ^2^/df < 3), root mean square error of approximation (RMSEA < 0.08), goodness-of-fit index (GFI > 0.90), Tucker-Lewis fit index (TLI > 0.90), and comparative fit index (CFI > 0.90) [41]. Poor fitting means that the model is not suitable for the data and needs to be modified. Based on the modification indices suggested by the AMOS, correlating error terms is a method to improve fitting when supported by a strong theoretical justification [41].

## Results

### Subject characteristics

The mean age of the sample was 46.3 (SD = 6.7, range = 27–60) years. The other characteristics of the sample are reported in Table 1.

**Table 1:** PCS, MCS and QOL scores of Chinese civil servants according to participant characteristics

	Number(%)	PCS		MCS		Total QOL	
		M (SD)	*P*	M (SD)	*P*	M (SD)	*P*
Gender							
Male	442 (79.07)	75.92 (15.41)	0.088	76.00 (16.96)	0.475	75.96 (15.18)	0.164
Female	105 (18.78)	73.12 (13.75)		74.89 (13.52)		74.01 (12.28)	
Missing value	12(2.15)						
Age (year)							
< 45	179 (32.02)	74.76 (15.98)	0.527	73.40 (16.78)	0.020	74.08 (15.40)	0.105
≥ 45	370 (66.19)	75.64 (14.91)		76.88 (16.12)		76.26 (14.43)	
Missing value	10(1.79)						
Number of years in the position (year)							
< 20	103 (18.43)	73.94 (16.67)	0.318	72.70 (17.70)	0.030	73.32 (16.27)	0.086
≥ 20	429 (76.74)	75.63 (15.06)		76.61 (16.06)		76.12 (14.48)	
Missing value	27(4.83)						
Marital status							
Married	533 (95.35)	75.60 (14.92)	0.025	76.05 (16.09)	0.005	75.83 (14.43)	0.007
Single/Divorced/Windowed	20(3.58)	67.88 (20.25)		65.63 (21.89)		66.75 (20.28)	
Missing value	6(1.07)						
Education level							
Associated degree^a^	27(4.83)	75.77 (13.52)	0.312	71.33 (17.85)	0.023	73.55 (14.24)	0.088
Bachelor degree	314 (56.18)	76.26 (15.14)		77.43 (16.34)		76.85 (14.71)	
Master degree or above	204 (36.49)	74.18 (15.37)		74.03 (15.98)		74.11 (14.69)	
Missing value	14(2.50)						
Total	559 (100.00)	75.31 (15.18)		75.68 (16.39)		75.49 (14.73)	

*M* mean, *SD* standard deviation

^a^Associated degree: It is a level of qualification between a high school diploma and a bachelor’s degree, which was obtained after completing 3 years of vocational education

### Univariate analyses of PCS, MCS and QOL

As shown in Table 1, the mean values of PCS, MCS, and total QOL were 75.31 (SD = 15.18), 75.68 (SD = 16.39), and 75.49 (SD = 14.73), respectively. Univariate analyses indicated significant differences in the PCS, MCS, and total QOL scores for marital status, and significant differences in MCS scores for age, number of years in the position, and education level. No significant differences in PCS, MCS, and total QOL scores were found for the other socio-demographic variables.

### Mean, standard deviations and correlations of the study variables

The means, standard deviations (SDs), and bivariate correlations are presented in Table 2. The Pearson’s correlation analysis positively correlated neuroticism with occupational stress, and negatively correlated it with job satisfaction and QOL. In addition, occupational stress was negatively correlated with job satisfaction and QOL, and job satisfaction was positively correlated with QOL.

**Table 2:** Mean, standard deviations and correlations among personality, occupational stress, Job satisfaction and QOL

Variables	M (SD)	Range	1	2	3	4
1. Neuroticism	19.75(6.01)	8.00–37.00	1			
2. Occupational stress	68.51 (11.94)	35.00–113.00	0.189**	1		
3. Job satisfaction	11.53(2.54)	3.00–15.00	−0.271**	− 0.226**	1	
4. QOL	75.49 (14.73)	12.50–100.0	− 0.450**	− 0.341**	0.357**	1

Range: the actual score range for each variable; ** *P* < 0.01

### Goodness-of-fit test of the hypothetical model and model modification

The results of the SEM analysis of the hypothetical model indicated that the data failed to support the theoretical model (see Table 3). Furthermore, the modification index indicated that three pairs of covariance parameters should be placed between MH and RE, PF and PR, and D2 and D7. As these relationships were consistent with theoretical considerations, covariance parameters were included in the model. In the final modified model, the data fit the model well (see Table 3).

**Table 3:** Goodness-of-fit statistics of the primary model and the modified models

Steps	Model description	P	χ^2^/df	GFI	CFI	TLI	RMSEA
1	Primary model	0.000	3.849	0.876	0.880	0.864	0.071
2	Add covariance between e21 and e22	0.000	3.360	0.891	0.901	0.887	0.065
3	Add covariance between e16 and e17	0.000	3.031	0.903	0.915	0.903	0.060
4	Add covariance between e8 and e13	0.000	2.818	0.913	0.925	0.913	0.057

e21: the error terms of MH (mental health, a item of SF-8); e22: the error terms of RE (role limitations due to emotional problems, a item of SF-8); e16: the error terms of PF (physical functioning, a item of SF-8); e17: the error terms of RP (role limitations due to physical health problems, a item of SF-8); e8: the error terms of D2 (the item “Is relaxed, and handles stress well” of Neuroticism subscale); e13: the error terms of D7 (the item “Remains calm in tense situations” of Neuroticism subscale)

*GFI* goodness-of-fit index, *CFI* comparative fit index, *TLI* Tucker-Lewis index, *RMSEA* root mean square error of approximation

### Analysis of the hypothetical model

The standardized estimates of the path coefficients for each variable are shown in Fig. 2. SEM revealed significant regression or correlation paths, and all beta path coefficients were statistically significant (*P* < 0.01).

### Mediation effect analysis of the hypothetical model

The results for the direct and indirect effects of neuroticism on QOL with occupational stress and job satisfaction as mediators are presented in Table 4. Neuroticism had a direct effect of 0.290 (*P* < 0.01) and an indirect effect of 0.070 (*P* < 0.01) on job satisfaction. Moreover, neuroticism had a direct effect and an indirect effect on QOL with a path coefficient of 0.402 (*P* < 0.01) and 0.124 (*P* < 0.01), respectively. In addition, occupational stress had a direct effect and an indirect effect on QOL with a path coefficient of 0.215 (*P* < 0.01) and 0.065 (*P* < 0.01), respectively. In general, the model explained 38% of the variance of QOL.

**Table 4:** The direct and indirect effects of neuroticism on QOL

Effects	Point estimate	95% bias-corrected CI
Effects of neuroticism on job satisfaction		
Direct effect	− 0.290	(− 0.393, − 0.180)**
Indirect effect	−0.070	(− 0.134, − 0.027)**
Effects of occupational stress on QOL		
Direct effect	−0.215	(− 0.338, − 0.082)**
Indirect effect	− 0.065	(− 0.116, − 0.030)**
Effects of neuroticism on QOL		
Direct effect	−0.402	(− 0.494, − 0.310)**
Indirect effect	−0.124	(− 0.187, − 0.072)**

**P < 0.01

## Discussion

The current study intended to investigate the QOL of Chinese civil servants and identify the paths of neuroticism affecting QOL. This study is meaningful, because it is the first analysis to explore the mediating role of occupational stress and job satisfaction in the relationship between neuroticism and QOL through an SEM analyses.

The scores for PCS, MCS, and total QOL in the present study were significantly higher than that found for young Chinese civil servants working in local government in a study by Lu and Liang [3]. A possible reason is that most participants in Lu and Liang’s study were aged less than 40 years, worked as staff, and were under pressure from being promoted and raising children.

The results of the univariate analyses associated PCS, MCS, and QOL with marital status. Consistent with a previous study [4], single civil servants might lack emotion and social support, which are related to lower QOL. In addition, civil servants who are older and have worked for more years had better mental health, perhaps because they have more work experience and the ability to deal with problems. Furthermore, the finding that MCS was associated with level of education suggested that civil servants’ mental health was more affected by education level than physical health. According to the results, civil servants with bachelor’s degrees had the highest MCS score. One possible reason is that civil servants with an associated degree may face more difficulties in being promoted, and those with master’s degrees or above may have higher requirements for themselves. Both these aspects may increase their anxiety and depression.

The most important goal of this study was to examine the relationships between neuroticism, occupational stress, job satisfaction, and QOL. As hypothesized, the results of the Pearson’s correlation analysis significantly related the four variables. In addition, the analysis of the structural model showed that neuroticism had both direct and indirect effects on QOL with occupational stress and job satisfaction acting as mediators.

To the best of our knowledge, this is the first study to combine the OSI-R model, JDC model, and ERI model to measure the level of professional stress. The study showed that the ORQ, JDC, and ERI could effectively reflect occupational stress. Therefore, this more comprehensive multi-dimensional approach was accurate and was a strength of this study. Employing the new measurement of occupational stress, this study confirmed that civil servants with higher occupational stress or lower job satisfaction experienced poor QOL, which was consistent with the findings of previous studies [12, 13]. More important, occupational stress indirectly affected QOL by decreasing job satisfaction. These results suggest that occupational stress and job satisfaction are crucial for the health of civil servants. The government could improve civil servants’ QOL by taking comprehensive measures to reduce occupational stress such as decreasing workload (e.g., reduce work outside of responsibilities, establish a regular coordination mechanism), improving the control of work (e.g., flexible work schedules, regular professional training to improve working ability), and promoting the balance between demands and rewards (e.g., adequate salary and respect). It can also improve occupational satisfaction by creating a comfortable working environment and supportive interpersonal relationships.

Previous studies related higher neuroticism with health status such as depressive symptoms, chronic diseases [42]. Consistent with previous studies [29], our data directly negatively correlated neuroticism with civil servants’ QOL. In addition, neuroticism had an indirect negative impact on QOL when mediated by occupational stress and job satisfaction. Here, the indirect effect sizes were small, but significant. This was consistent with existing literature that explained the relatively small but significant effects of neuroticism on job strain and job satisfaction [33]. Considering the stability of personality traits in healthy adulthood, personality, especially neuroticism, should be measured in job interviews, and it is reasonable to select individuals with a low level of neuroticism as civil servants.

Three pairs of covariance parameters were included in the final model, as these relationships were consistent with theoretical considerations; for example, the correlation of error terms for PF (“During the past four weeks, to what extent did physical health problems limit your physical activities (such as walking or climbing stairs)?”) and RP (“During the past four weeks, how much difficulty did you experience doing your daily work, both at home and away from home, because of your physical health?”). If a person’s physical health problems limit their physical activity, indicating that the person’s physical health is poor, then the person does not have the physical strength and energy to complete their daily work, and vice versa. Therefore, these two items were closely associated.

There are some limitations in this study. First, a cross-sectional design was used. Therefore, the causal relationship between neuroticism, occupational stress, job satisfaction, and QOL cannot be inferred, and should be confirmed in further prospective studies. Second, we only explored the mediating effects of two mediators in the relationship between neuroticism and QOL in this study. In addition to the pathway shown in our study, other factors might mediate the relationship between neuroticism and QOL, such as emotion regulation [43], coping style, and social support [28]. The mediating effects found for other staff should be verified among civil servants in a future study. Last, all participants were from Shandong Province, and the sample was not randomized, which suggested the generalization of results to other areas of China is limited.

## Conclusions

In conclusion, the participants had high self-reported QOL levels. Neuroticism, occupational stress, and job satisfaction explained 38% of the variance in QOL, and occupational stress and job satisfaction mediated the relationship between neuroticism and QOL. These results not only facilitate further understanding of the association of neuroticism, occupational stress, job satisfaction, and QOL, but also provide directions for interventions to improve civil servants’ health. Considering the strong effect of neuroticism on QOL, a personality assessment could be considered in civil servant recruitment. In addition, measures could be taken to reduce occupational stress and increase job satisfaction to improve the health of civil servants, especially those who are neurotic.

## Data Availability

The datasets used and analysed during the current study are available from the corresponding author on reasonable request.

## Abbreviations

BP: Bodily pain
CFA: Confirmatory factor analysis
CFI: Comparative fit index
ERI: Effort-reward imbalance
GFI: Goodness-of-fit index
GH: General health perceptions
JDC: Job demand-control
MCS: Mental component summary
MH: Mental health
ORQ: Occupational Role Questionnaire
OSI-R: Occupational Stress Inventory-Revised
PCS: Physical component summary
PF: Physical functioning
QOL: Quality of life
RE: Role limitations due to emotional problems
RMSEA: Root mean square error of approximation
RP: Role limitations due to physical health problems
SEM: Structural equation modeling
SF: Social functioning
SF-8: Short Form-8 Health Survey
TLI: Tucker-Lewis fit index
VT: Vitality
χ^2^/df: Normed Chi-square
